# Dietary Quercetin Supplementation in Mice Increases Skeletal Muscle *PGC1α* Expression, Improves Mitochondrial Function and Attenuates Insulin Resistance in a Time-Specific Manner

**DOI:** 10.1371/journal.pone.0089365

**Published:** 2014-02-21

**Authors:** Tara M. Henagan, Natalie R. Lenard, Thomas W. Gettys, Laura K. Stewart

**Affiliations:** 1 Department of Nutrition Science, Purdue University, West Lafayette, Indiana, United States of America; 2 Department of Sciences, Our Lady of the Lake College, Baton Rouge, Louisiana, United States of America; 3 Laboratory of Nutrient Sensing and Adipocyte Signaling, Pennington Biomedical Research Center, Baton Rouge, Louisiana, United States of America; 4 School of Kinesiology, Louisiana State University, Baton Rouge, Louisiana, United States of America; Pontificia Universidad Catolica de Chile, Chile

## Abstract

**Aims/Hypothesis:**

High fat diet (HFD)-induced insulin resistance (IR) is partially characterized by reduced skeletal muscle mitochondrial function and peroxisome proliferator activated receptor gamma coactivator 1 alpha (PGC1α) expression. Our previous study showed that a high dose of the bioflavonoid quercetin exacerbated HFD-induced IR; yet, others have demonstrated that quercetin improves insulin sensitivity. The aim of this study was to investigate whether differing doses of quercetin act in a time-dependent manner to attenuate HFD-induced IR in association with improved skeletal muscle mitochondrial function and *PGC1α* expression.

**Methods:**

C57BL/6J mice were fed HFD for 3 or 8 wks, with or without a low (50 ug/day; HF+50Q) or high (600 ug/day, HF+600Q) dose of quercetin. Whole body and metabolic phenotypes and insulin sensitivity were assessed. Skeletal muscle metabolomic analysis of acylcarnitines and *PGC1α* mRNA expression via qRT-PCR were measured.

**Results:**

Quercetin at 50 ug/day for 8 wk attenuated HFD-induced increases in fat mass, body weight and IR and increased *PGC1α* expression, whereas 600 ug/day of quercetin exacerbated fat mass accumulation without altering body weight, IR or *PGC1α*. *PGC1α* expression correlated with acylcarnitine levels similarly in HF and HF+600Q; these correlations were not present in HF+50Q. At both time points, energy expenditure increased in HF+50Q and decreased in HF+600Q, independent of *PGC1α* and IR.

**Conclusions/Interpretation:**

Chronic dietary quercetin supplementation at low but not higher dose ameliorates the development of diet-induced IR while increasing *PGC1α* expression in muscle, suggesting that skeletal muscle may be an important target for the insulin-sensitizing effects of a low dose of quercetin.

## Introduction

Reduced skeletal muscle mitochondrial function may play an integral role in the onset of insulin resistance (IR) during diet-induced obesity [1]–[5]. Integration of dietary fat intake and alterations in mitochondrial biogenesis and function are also partially mediated by peroxisome proliferator receptor gamma coactivator 1 alpha (PGC1α) expression [6]–[8]. *PGC1α* is a transcriptional coactivator that coordinates gene expression from the mitochondrial and nuclear genomes to change in mitochondrial number or function [9]–[11]. In skeletal muscle, *PGC1α* plays an important role in fiber type switching to oxidative type 1 fibers [12] and in promoting mitochondrial biogenesis [11]. Importantly, *PGC1α* is downregulated in skeletal muscle by HFD and decreased in skeletal muscle of type 2 diabetic individuals [6],[13]. The downregulation of *PGC1α* expression and mitochondrial function in response to diet makes *PGC1α* an ideal target for the treatment of obesity and type 2 diabetes.

Quercetin, a bioflavonoid abundant in apples, onions and tea, is a powerful antioxidant that has the potential to improve insulin action [14] and skeletal muscle mitochondrial biogenesis [15]. For example, 6 wks of a quercetin diet (0.08% and 0.04%) significantly reduced plasma glucose levels in obese, leptin receptor deficient C57BL/KsJ-db/db mice [16] and improved hyperinsulinemia in obese Zucker rats [17]. Dietary quercetin has also been shown to increase skeletal muscle *PGC1α* and mitochondrial number [18]. In contrast, we previously found that a higher dose of dietary quercetin exacerbated HFD-induced IR while increasing long chain but not short chain acylcarnitines, suggestive of incomplete beta oxidation and may indicate reduced mitochondrial function in skeletal muscle in C57BL6/J mice [19]. Given that others have also found contradictory effects of quercetin, with seemingly higher doses being detrimental to cellular systems [20]–[22], we hypothesized that dietary quercetin supplementation acts in a time- and dose-dependent manner to affect IR in association with alterations in skeletal muscle *PGC1α* gene expression and mitochondrial function. Consequently, we administered dietary quercetin in a low or high dose in conjunction with a HFD and examined phenotypic alterations, whole body insulin sensitivity and metabolism. In addition, we determined whether quercetin altered skeletal muscle *PGC1α* and acylcarnitine profiles in a time- and dose-dependent manner.

## Methods

### Animals

All experiments were reviewed and approved by the Pennington Biomedical Research Center Institutional Animal Care and Use Committee. Male C57BL/6J mice (N = 48) (stock number 000664, Jackson Laboratory, Bar Harbor, ME, USA) were obtained and weaned onto a low fat diet (10% kcal fat, Research Diets, New Brunswick, NJ, USA). At 6 wks of age, mice (N = 16 per group) were randomly assigned to one of the following diets (Research Diets): 1. high fat (HF; 45% kcal fat; D12451), 2. high fat+high dose of quercetin (HF+600Q; 45% kcal fat +600 ug/mouse/day; D08072303) or high fat+low dose of quercetin (HF+50Q; 45% kcal fat +50 ug/mouse/day; D08072305). All mice were singly housed in shoebox cages with corncob bedding in controlled environmental conditions (22°C) on a 12 hour light:dark cycle. At 3 wks and 8 wks after feeding respective diets, mice were euthanized and quadriceps muscle were harvested and snap frozen in liquid nitrogen for measurement of gene expression or metabolomic analysis.

### Diets, Food Consumption and Phenotyping

Quercetin (Q0125) was purchased from Sigma Aldrich (St. Louis, MO, USA) and HF+Q diets were cold processed by Research Diets. All diets were stored at 4°C in light-protective, airtight containers. Food was changed every 3 d and water was provided *ad libitum.* Food consumption was assessed weekly by weighing food before and after a 48 h period. Body weight and composition (NMR Bruker Minispec, Billerica, MA, USA) were measured weekly for the duration of the study. Percent fat and percent (LBM) were calculated by dividing fat mass or LBM by total body weight and multiplying by one hundred.

### Indirect Calorimetry

Energy expenditure (N = 8 per group) was evaluated at 3 wks and 7 wks after feeding the respective diets. Mice were housed individually in metabolic chambers (Oxymax Comprehensive Lab Animal System; Columbus Instruments, Columbus, OH, USA) on a 12 h light-dark cycle. After a 24 h acclimation period, the volume of oxygen consumption (VO2) and volume of carbon dioxide production (VCO2) was measured for 72 h. Respiratory exchange ratio (RER) was calculated from VCO2 and VO2. Energy expenditure was calculated from VO2 and RER measurements (VO2 X (3.815+ (1.23 X RER)) X 40.1868) and expressed as kJ per kg body weight (BW) per hour (Flowmax, Columbus Instruments, Columbus, OH, USA). Ambulatory and total physical activity, measured by beam breaks, was also obtained during this time.

### Insulin Sensitivity

Insulin sensitivity was assessed by insulin tolerance test (ITT) and fasting blood glucose measurements. Mice were fasted for 4 h and blood glucose was measured with a One Touch Basic glucometer (Milpitas, CA, USA). After measuring fasting blood glucose, mice were injected intraperitoneally with 0.75 U/kg BW of insulin (NovolinR, Nordisk, Bagsvaerd, Denmark). Blood glucose measurements were made 15, 30 60 and 120 minutes following injection. Area under the curve (AUC) was calculated for individual mice using GraphPad Prism 5.0 and averaged for each treatment group.

### qRT-PCR

Total RNA was extracted from quadriceps muscle tissue using Tri-Reagent (Molecular Research Center, Cincinnati, OH, USA) followed by further purification with a RNeasy mini kit and RNase-free DNase (Qiagen, Valencia, CA, USA). Degradation of isolated RNA was prevented by addition of SUPERase-In (Ambion, Austin, TX, USA). The quantity and quality of the RNA was analyzed by spectrophotometry (ND-1000, NanoDrop Technologies, Wilmington, DE, USA) and confirmed by gel electrophoresis. RNA was reverse transcribed into a cDNA library using M-MLV reverse transcriptase (Promega, Madison, WI, USA). qRT-PCRwas performed using specific primers targeted towards exon-exon junctions that were designed using Primer-Express v2.0.0 software (Applied Biosystems, Foster City, CA, USA). All samples were run in duplicate on the SmartCycler platform (Cepheid, Sunnyvale, CA, USA). *PGC1α* gene expression was analyzed using a standard curve and normalization to cyclophilin B, an endogenous control.

### Metabolomics

Tandem mass spectrometry analysis was used to evaluate muscle extracts for 45 acylcarnitine (AC) species ranging from 2 to 20 carbons, in saturated and unsaturated forms as previously described [19]. Briefly, protein extracts were prepared from tissues extracted at the 4 wk and 8 wk time points at the zenith and nadir of the metabolic cycle. Quadriceps muscle extracts were prepared by pulverizing 50 µg of frozen tissue in liquid nitrogen_,_ cold ddH_2_0 was added and the samples were homogenized on ice. Samples were subjected to 1 freeze-fracture cycle, sonicated and centrifuged. The muscle supernatant was stored at −80°C until further analysis by tandem mass spectrometry on the SYNAPT HDMS platform (Waters Corporation, Milford, MA, USA).

### Statistical Analysis

The data were analyzed with JMP statistical analysis software and results are expressed as means ± standard error. Body composition parameters and glucose tolerance test (GTT) were analyzed by repeated measures ANOVA. All other measurements were analyzed by 3(diet) X 2(time point) ANOVA. A Tukey test was used post hoc as necessary. A P value <0.05 was used to determine significance.

## Results

### Whole Body Phenotypes

**Figure 1:**
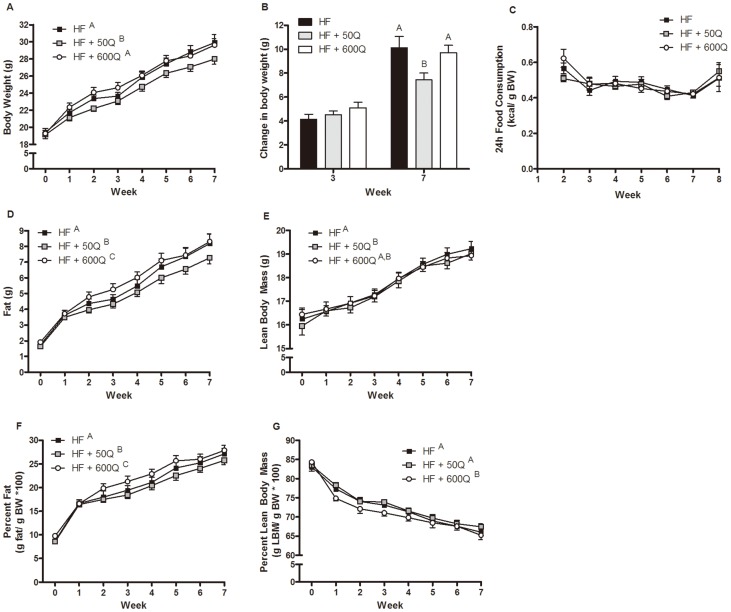
Body weight was measured weekly in mice fed a HFD (HF; black), HFD plus 50 ug/day quercetin (HF+50Q; gray) or a HFD plus 600 ug/day of quercetin (HF+600Q; white) and is presented as means ± SEMs (A) and as the change in body weight over the course of the study (B). (**C**) Weekly food intake was measured over a 48 h period and is shown as the average 24 h food consumption. Body composition was assessed by NMR weekly and is reported as fat mass (**D**), lean body mass (**E**), percent fat (**F**) and percent lean body mass (**G**). All values are reported as means ± SEM. Significant differences between groups by a post hoc Tukey test is denoted by a difference in superscript.

HF+50Q decreased BW compared to HF, whereas HF+600Q showed no difference in BW compared to HF (Fig. 1A). By week 8 of the study, HF exhibited a 10.11 g increase in BW compared to week 0, HF+50Q increased by 7.45 g and HF+600Q increased by 9.71 g (Fig. 1B). Despite significant differences in BW, there were no significant differences in food consumption among the groups any groups (Fig. 1C). Differences in BW between HF and HF+50Q appeared to result predominantly from significant changes in fat mass (Fig. 1D), with HF+50Q significantly decreasing fat mass over the course of the 8 week supplementation period (Fig. 1D). However, LBM was also decreased slightly but significantly in HF+50Q compared to HF (Fig. 1E). Although BW was not significantly different between HF and HF+600Q, there was a significant increase in fat mass between the two groups, which converged by the end of the 8 wk period (Fig. 1D). Likewise, percent body fat was significantly lower in HF+50Q but higher in HF+600Q (Fig. 1F); whereas, percent LBM was not different between HF and HF+50Q but decreased in HF+600Q (Fig. 1G).

### Metabolic Phenotype and Insulin Sensitivity

**Figure 2:**
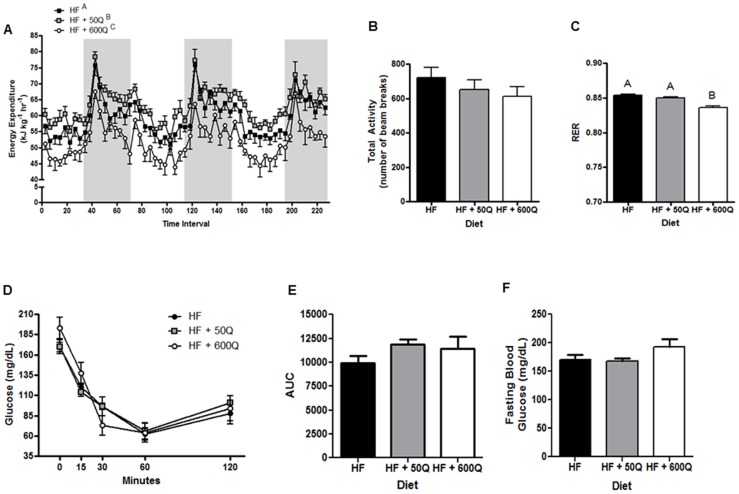
Energy expenditure (A), physical activity (B) and respiratory exchange ratio (RER) (C) were measured by indirect calorimetry in C57BL/6J mice after 3 wks of feeding respective diets. Gray bars represent night periods in A. Significant differences between groups are denoted by differing superscripts. Insulin sensitivity was assessed by measuring blood glucose levels before and during an insulin tolerance test after 3 wks on respective diets. Data are presented before, and at 15, 30, 60 and 120 mins after injection of insulin (**D**) and as the area under the curve (AUC) for each treatment group (**E**). (**F**) Fasting blood glucose levels are also reported. All values are shown as the mean ± SEM.

Metabolic phenotype was assessed in all groups of mice after 3 wks and 7 wks on the diets. At 3 wks, energy expenditure was significantly increased in HF+50Q but decreased in HF+600Q compared to HF (Fig. 2A) (Table S1), despite no differences in physical activity among the groups (Fig. 2B). HF+600Q significantly decreased RER, indicating a greater reliance on fatty acid as a metabolic substrate in this group, whereas the lower dose of had no effect on RER (Fig. 2C). In addition, there were no differences among groups at 3 wks in whole body insulin sensitivity, assessed by ITT (Fig. 2D and E) or fasting blood glucose levels (Fig. 2F).

**Figure 3:**
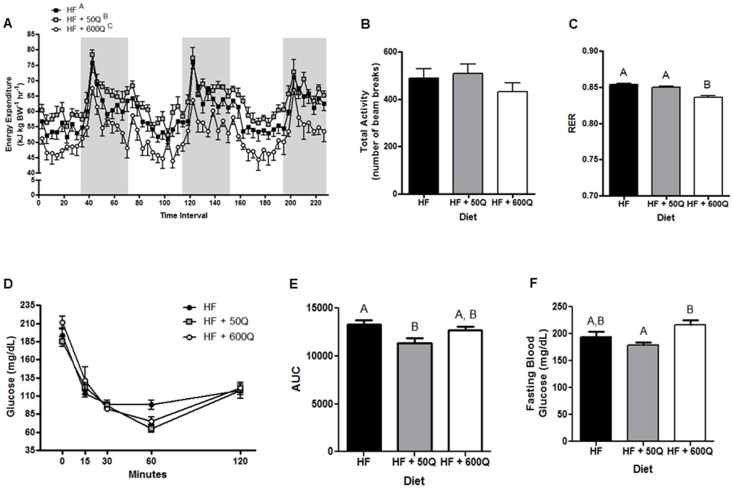
After 8 wks of feeding respective diets, energy expenditure (A), physical activity (B) and respiratory exchange ratio (RER) (C) were measured by indirect calorimetry. Gray bars represent night periods in A. Insulin sensitivity was assessed by an insulin tolerance test after 8 wks on respective diets and is shown as the average blood glucose levels before, and at 15, 30, 60 and 120 mins after injection of insulin (**D**) and as the average area under the curve (AUC) for each treatment group (**E**). (**F**) Fasting blood glucose levels are also reported. All values are shown as the mean ± SEM. Significant differences between groups are denoted by differing superscripts.

The pattern of effects due to dietary quercetin supplementation on energy expenditure, activity levels and RER remained constant from 3 wk to 8 wks (Fig. 3A–C) (Table S1). However, the ITT showed that the low dose of quercetin was able to improve whole body insulin sensitivity at 8 wks when compared to HF (Fig. 3D); whereas, the higher dose of quercetin had no effect on HFD-induced changes in insulin tolerance (Fig. 3E). Additionally, HF+50Q but not HF+600Q experienced a decrease in plasma blood glucose levels at 8 wks, although this decrease did not reach statistical significance compared to HF but did when compared to HF+600Q (Fig. 3F).

### Acylcarnitines

**Figure 4:**
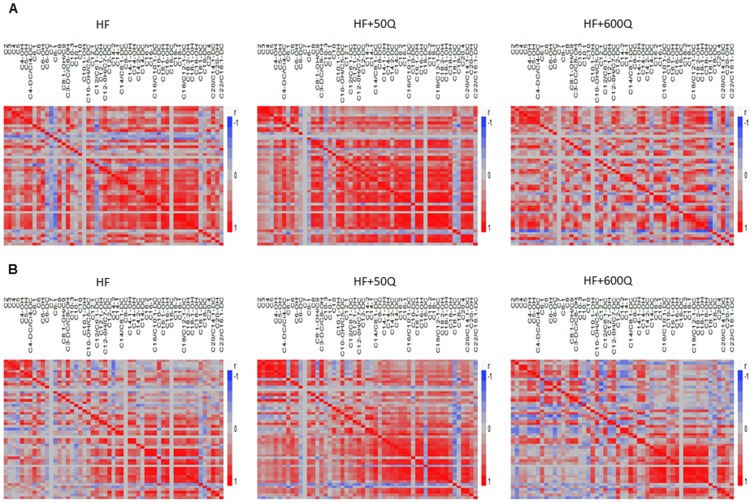
Acylcarnitines were measured in quadriceps muscle after 3 wks (A) and 8 wks (B) in HF, HF+50Q and HF+600Q, and pairwise correlation matrices were generated. Red represents a +1 correlation and blue a −1. Acylcarnitine species are named at the top of each matrix, with the iteration of acylcarnitines occurring vertically.

Acylcarnitine species (45) were measured in skeletal muscle tissue extracts from mice after 3 wks and 8 wks of feeding and are presented in Table 1. Pairwise correlation matrices were generated for all acylcarnitine levels within each treatment group and time point. Surprisingly, given the changes in energy expenditure at 3 wks, there were relatively small differences in correlation matrices between any groups at this time point (Fig. 4A). However, the most notable difference in matrices appeared to occur between HF and HF+600Q, with HF and HF+50Q being similar (Fig. 4A). Both HF and HF+600Q but not HF+50Q experienced a decrease in the number of strong positive correlations between medium chain species (Fig. 4A). Additionally, HF+600Q exhibited fewer strong positive and more negative correlations between long and short chain species (Fig. 4A). However, by 8 wks of supplementation strong positive correlations that were present between long and medium chain acylcarnitines were diminished in all treatment groups compared to correlations seen at 3 wks (Fig. 4A and B). In both HF and HF+600Q, there were more negative correlations present between long and medium and long and short chain species. When comparing acylcarnitine correlations across time, the low dose of quercetin supplementation prevented weakening of these positive correlations from 3 wks to 8 wks, specifically those between the long and short chain acylcarnitines (Fig. 4A and B), the high dose of quercetin supplementation exaggerated the number of strong correlations between acylcarnitine species being reversed or diminished compared to HF alone (Fig. 4A and B).

**Table 1:** Acylcarnitines.

Acylcarnitine	Time	HF	HF+50Q	HF+600Q
CN	3 wk	2.464570±0.359200	2.141595±0.314587	2.198981±0.417009
	8 wk	2.891080±0.339958	2.974340±0.403685	2.669783±0.283541
C2	3 wk	2.303210±0.276210	2.107605±0.313589	2.278700±0.402551
	8 wk	3.275060±0.398258	3.325075±0.429395	3.250083±0.335423
C3	3 wk	0.010435±0.001668	0.008920±0.002155	0.011350±0.001360
	8 wk	0.012707±0.002138	0.011735±0.002724	0.011606±0.002299
C4	3 wk	0.067935±0.005913	0.073120±0.012329	0.056513±0.007517
	8 wk	0.092985±0.011244	0.079390±0.010965	0.076328±0.007917
C5	3 wk	0.011970±0.001327	0.017270±0.004532	0.009963±0.002122
	8 wk	0.021833±0.005936	0.020170±0.004247	0.019956±0.003415
C4-OH	3 wk	0.036580±0.006009	0.028235±0.004595	0.030588±0.006332
	8 wk	0.045498±0.006922	0.035915±0.004305	0.035950±0.005033
C5-OH	3 wk	0.024520±0.003348	0.020210±0.002669	0.019550±0.003352
	8 wk	0.025425±0.003120	0.029005±0.003885	0.023272±0.002862
C4-DC/Ci4-DC	3 wk	0.013135±0.001653	0.012190±0.002626	0.013981±0.002639
	8 wk	0.024955±0.004453	0.020515±0.003324	0.022722±0.004376
C6∶1	3 wk	0.002100±0.000310	0.001855±0.000193	**0.003175±0.000291** *
	8 wk	0.002540±0.000331	0.002515±0.000373	**0.004389±0.000542** *
C6	3 wk	0.012110±0.001450	0.009235±0.001258	0.008388±0.001137
	8 wk	0.015558±0.001415	0.013920±0.001773	**0.010344±0.000972**
C6-OH	3 wk	0.000570±0.000304	0.000600±0.000201	0.000413±0.000188
	8 wk	0.000878±0.000187	0.001560±0.000518	0.003922±0.003378
C6-DC	3 wk	0.001265±0.000307	0.000845±0.000217	0.000513±0.000099
	8 wk	0.000820±0.000158	0.000710±0.000247	0.000656±0.000083
C7	3 wk	0.000010±0.000010	0.000000±0.000000	0.000000±0.000000
	8 wk	0.000000±0.000000	0.000000±0.000000	0.000272±0.000272
C8-1	3 wk	0.004750±0.000326	0.005360±0.000287	**0.007044±0.000435** *
	8 wk	0.005652±0.000361	0.006450±0.000418	**0.008622±0.000483** *
C8	3 wk	0.002610±0.000840	0.002525±0.000854	0.002188±0.000581
	8 wk	0.004455±0.000862	0.005435±0.000948	**0.002628±0.000615**
C8∶1-OH/C9	3 wk	0.000100±0.000052	0.000100±0.000100	0.000081±0.000063
	8 wk	0.000075±0.000056	0.000060±0.000028	0.000067±0.000045
C3-DC/C8-OH	3 wk	0.010380±0.001547	0.007680±0.001333	0.009181±0.001631
	8 wk	0.013750±0.001657	0.013310±0.001911	0.014411±0.002379
C10-3	3 wk	0.000755±0.000643	0.000250±0.000244	0.000150±0.000063
	8 wk	0.000040±0.000040	0.000065±0.000043	0.000167±0.000113
C10∶1	3 wk	0.003040±0.000405	0.003485±0.000234	**0.004631±0.000366** *
	8 wk	0.003878±0.000147	0.003790±0.000313	**0.004606±0.000293**
C10	3 wk	0.001305±0.000335	0.001555±0.000430	0.001206±0.000345
	8 wk	0.000938±0.000442	0.000600±0.000281	0.000472±0.000255
C10∶1-OH	3 wk	0.000000±0.000000	0.000010±0.000010	0.000000±0.000000
	8 wk	0.000015±0.000015	0.000040±0.000018	0.000050±0.000035
C10-OH/C5-DC	3 wk	0.002185±0.000391	0.001485±0.000352	0.001594±0.000348
	8 wk	0.003520±0.000526	0.003055±0.000386	0.002761±0.000606
C12∶1	3 wk	0.004110±0.000117	0.004125±0.000140	**0.004919±0.000259** *
	8 wk	0.004127±0.000159	0.004090±0.000141	**0.004772±0.000156** *
C12/C6∶1-DC	3 wk	0.001530±0.000128	0.001315±0.000236	0.001150±0.000254
	8 wk	0.000968±0.000215	0.001120±0.000333	0.000800±0.000315
C12∶1-OH	3 wk	0.000255±0.000089	0.000150±0.000067	0.000056±0.000032
	8 wk	0.000510±0.000170	0.000370±0.000091	0.000411±0.000195
C12-0H/C7-DC	3 wk	0.001895±0.000423	0.001005±0.000329	0.001344±0.000376
	8 wk	0.002877±0.000410	0.002370±0.000415	0.002400±0.000589
C12-DC	3 wk	0.002075±0.000481	0.002075±0.000333	0.001825±0.000422
	8 wk	0.003418±0.000668	0.004550±0.000922	0.003961±0.001000
C14-2	3 wk	0.000385±0.000115	0.000345±0.000204	0.000244±0.000089
	8 wk	0.000317±0.000091	0.000380±0.000136	0.000217±0.000084
C14-1	3 wk	0.002235±0.000202	0.001870±0.000370	0.001394±0.000360
	8 wk	0.002513±0.000453	0.002405±0.000570	0.001194±0.000388
C14/C8∶1-DC	3 wk	0.004645±0.000860	0.003570±0.000536	0.002606±0.000538
	8 wk	0.006283±0.001052	0.007470±0.001169	0.004500±0.001135
C8-DC	3 wk	0.000010±0.000010	0.000020±0.000015	0.000013±0.000013
	8 wk	0.000000±0.000000	0.000010±0.000010	0.000039±0.000022
C14-1-OH	3 wk	0.002250±0.000449	0.001180±0.000398	0.001256±0.000281
	8 wk	0.004090±0.000545	0.003080±0.000510	0.002978±0.000681
C14-OH	3 wk	0.001990±0.000292	0.001150±0.000288	0.001344±0.000249
	8 wk	0.003017±0.000353	0.002795±0.000328	0.002578±0.000465
C14∶2-DC	3 wk	0.001610±0.000365	0.000710±0.000204	0.000969±0.000300
	8 wk	0.002130±0.000609	0.002225±0.000491	0.001778±0.000236
C14-DC	3 wk	0.000165±0.000071	0.000100±0.000032	0.000100±0.000033
	8 wk	0.000130±0.000033	0.000330±0.000074	0.000361±0.000125
C16∶2	3 wk	0.002425±0.000463	0.001920±0.000493	0.001819±0.000399
	8 wk	0.003540±0.000544	0.004590±0.000580	0.002928±0.000547
C16-1	3 wk	0.005800±0.001428	0.004720±0.001111	0.002975±0.000566
	8 wk	0.007880±0.001334	0.010190±0.001743	0.005917±0.001124
C16/C10∶1-DC	3 wk	0.025385±0.007684	0.019080±0.005025	0.011613±0.002322
	8 wk	0.030255±0.005623	0.046220±0.007572	0.032994±0.009475
C10-DC	3 wk	0.015260±0.005301	0.016425±0.004833	0.012119±0.004124
	8 wk	0.011720±0.006513	**0.028035±0.004823** *	0.011311±0.002841
C16∶1-OH	3 wk	0.002475±0.000417	0.001820±0.000470	0.001875±0.000344
	8 wk	0.003843±0.000466	0.004025±0.000622	0.003522±0.000567
C16-OH	3 wk	0.001945±0.000381	0.001375±0.000430	0.001500±0.000283
	8 wk	0.003312±0.000560	0.004025±0.000663	0.003506±0.000553
C16-DC	3 wk	0.000000±0.000000	0.000000±0.000000	0.000006±0.000006
	8 wk	0.000000±0.000000	0.000015±0.000011	0.000000±0.000000
C18-2	3 wk	0.006795±0.001936	0.007210±0.002701	0.003769±0.000786
	8 wk	0.007565±0.001419	**0.011500±0.001968**	0.006111±0.001312
C18-1	3 wk	0.024015±0.006711	0.020945±0.006635	0.012100±0.002595
	8 wk	0.029340±0.005812	0.042495±0.007508	0.027394±0.006660
C18/C12-1-DC	3 wk	0.013955±0.002666	0.011360±0.002363	0.009275±0.001488
	8 wk	0.018107±0.003024	0.025210±0.003960	0.020328±0.004892
C18∶2-OH	3 wk	0.001765±0.000283	0.001555±0.000535	0.001438±0.000247
	8 wk	0.002798±0.000407	0.003000±0.000468	0.002522±0.000320
C18-1-OH	3wk	0.003100±0.000625	0.002650±0.000715	0.002100±0.000533
	8 wk	0.005450±0.000885	0.005960±0.000978	0.005078±0.000960
C18-OH	3wk	0.000725±0.000140	0.000615±0.000191	0.000631±0.000173
	8 wk	0.001565±0.000296	0.001950±0.000342	0.001422±0.000251
C18∶1-DC	3 wk	0.000035±0.000017	0.000085±0.000026	0.000056±0.000018
	8 wk	0.000030±0.000021	0.000050±0.000020	0.000039±0.000027
C18-DC	3 wk	0.000055±0.000014	0.000030±0.000020	0.000069±0.000025
	8 wk	0.000015±0.000008	**0.000080±0.000019** *	0.000056±0.000021
C20∶4	3 wk	0.000205±0.000126	0.000200±0.000092	0.000238±0.000148
	8 wk	0.000237±0.000064	**0.000570±0.000179**	0.000206±0.000083
C20/C14∶1-DC	3 wk	0.001185±0.000258	0.000755±0.000234	0.000669±0.000158
	8 wk	0.001438±0.000348	0.001795±0.000345	0.001900±0.000526
C20-OH	3 wk	0.000065±0.000029	0.000035±0.000018	0.000013±0.000013
	8 wk	0.000088±0.000027	0.000100±0.000029	0.000061±0.000022
C22/C16∶1-DC	3 wk	0.000005±0.000005	0.000005±0.000005	0.000019±0.000019
	8 wk	0.000013±0.000013	0.000020±0.000013	**0.000094±0.000039** *

Acylcarnitine values are shown in µM.

*denotes a significant difference (P<0.05) from HF by post hoc Tukey test. Significantly different and trending (P<0.081) values from HF are bolded.

Consistent with the correlation matrices results, we observed several significantly increased acylcarnitine species in HF+600Q compared to HF, including C8-1, C10∶1 and C12∶1, but no differences between HF and HF+50Q at 3wks (Table 1). At 8wks, these differences between HF and HF+600Q remained in all species except C10∶1 (Table 1). Additionally, HF+600Q showed significant increases in C6∶1 and C22/C16∶1-DC compared to HF and HF+50Q (Table 1). HF+50Q experienced significant differences compared to both HF and HF+600Q in medium and long chain acylcarnitine species, increasing in C10-DC compared to HF and HF+600Q and in C18-DC compared to HF but not HF+600Q (Table 1). Several other species also showed trends in changes between groups (P<0.081), including a decrease in C6 and C8 and an increase in C10∶1 in HF+600Q compared to HF and HF+50Q and an increase in C18-2 and C20∶4 in HF+50Q compared to both HF and HF+600Q.

### PGC1α Expression

**Figure 5:**
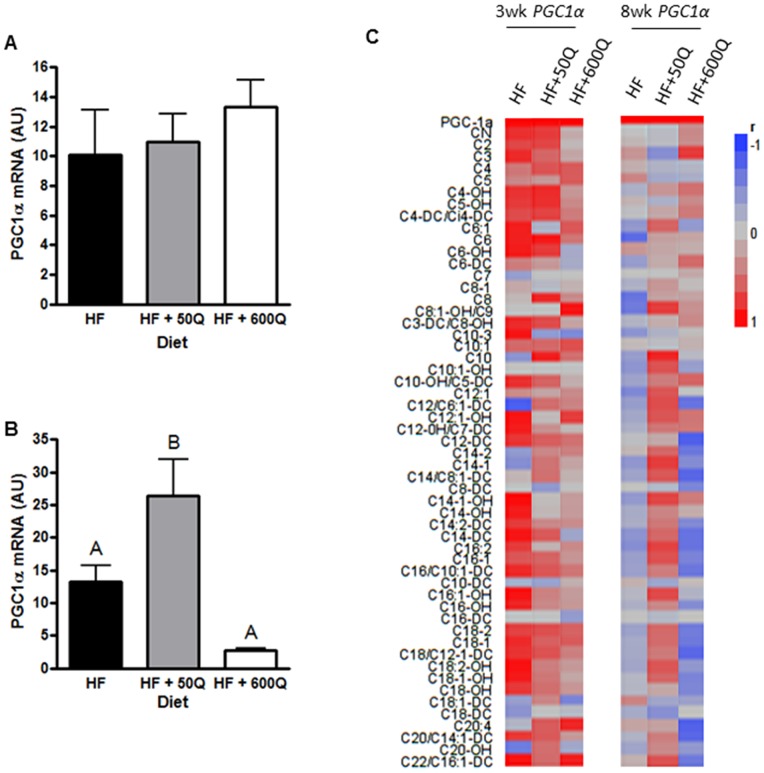
*PGC1α* mRNA was quantified by qRT-PCR after 3wks (A) and 8wks (B) of feeding respective diets and is shown as means ± SEMs in arbitrary units (AU) for each group. Significant differences by Tukey post hoc are denoted by differing superscripts. (**C**) *PGC1α* mRNA levels were correlated with acylcarnitine levels in each group after 3 and 8wks of feeding in HF, HF+50Q or HF+600Q. −1 correlations between gene expression and an acylcarnitine is represented by blue and +1 correlations by red.

*PGC1α* mRNA expression was measured in skeletal muscle after 3wks and 8wks of in all treatment groups. There were no differences in *PGC1α* mRNA expression among the groups at 3wks (Fig. 5A). However, at 8wks, the lowest dose of quercetin was able to significantly increase *PGC1α* mRNA expression compared to HFD (Fig. 5B). Although the high dose of quercetin decreased *PGC1α* at 8wks compared to HF, this decrease was not statistically significant (Fig. 5B). The strongest correlations were seen between *PGC1α* and short, medium, and long acylcarnitines in the HF group compared to the others (Fig. 5C). HF+50Q maintained a strong relationship between *PGC1α* and short chain acylcarnitines, but decreased the strength of correlations with long and medium chain acylcarnitines to *PGC1α* expression when compared to HF (Fig. 5C). HF+600Q exhibited even further decreases in correlation strength or no correlation in all chain lengths with *PGC1α* expression (Fig. 5C). When comparing correlation matrices of acylcarnitines with *PGC1α* expression among dietary groups at 3wks, HF and HF+50Q showed similar correlation patterns in short and long chain species, and HF and HF+600Q showed similar correlation patterns in medium chain species (Fig. 5C). Interestingly, the strong positive correlations between *PGC1α* and acylcarnitines revealed at 3wks in HF were almost totally reversed by 8wks of feeding to produce negative correlations (Fig. 5C). This reversal was exacerbated in the HF+600Q group, specifically in the long and medium chains (Fig. 5C). Yet, consistent with the insulin sensitizing effects of the low quercetin dose, HF+50Q maintained a similar level and strength in most positive correlations in all chain lengths at 8wks as that seen in the same group at 3wks (Fig. 5C).

## Discussion

Our previous work shows that a high dose of quercetin (24 mg/day) in conjunction with a HFD exacerbates whole body IR after 3wks of feeding, and this alteration is abolished after 8wks of supplementation [19]. However, others have shown a beneficial effect of quercetin at lower doses, ranging from 0.8 mg/day to 7.2 mg/day and after 7 days to 10wks of supplementation in rodents [17],[23],[24]. The insulin sensitizing effects of quercetin have been tested in several rodent models, including streptozocin-induced diabetic rats and genetic mouse models of impaired leptin signaling, db/db and ob/ob mice [17],[23]–[25]. Yet, it is important to note that the development of IR and type 2 diabetes in humans is due mostly to environmental conditions, such as low physical activity and HFD [26],[27]. Although our preliminary study in HFD-induced obese C57BL/6J mice showed that quercetin supplementation exaggerated IR [19], our previous results were based on supplementation of quercetin at a level at least 3 times higher than other groups [17],[19],[23]–[25]. Thus, the conflict of our findings with other reports suggests that quercetin may act in a time- and dose-dependent manner to affect insulin sensitization.

Several factors contribute to the pathogenesis of HFD-induced IR and, in one case, HFD-induced IR occurs independent of decreases in skeletal muscle mitochondrial function [28]. HFD- induced decreases in energy expenditure and skeletal muscle mitochondrial function may play an important and causative role in the onset of IR and type 2 diabetes [29]. This study demonstrates that 3wks of 50 ug/day of dietary quercetin supplementation increases energy expenditure without altering BW or composition. After 8wks of supplementation, this lower dose of quercetin attenuates HFD-induced IR, improves BW and fat gain, maintains increased energy expenditure and improves skeletal muscle mitochondrial function. Conversely, 3wks of quercetin supplementation at 600 ug/day causes the opposite effect, decreasing energy expenditure and slightly altering the acylcarnitine correlation matrix, which is indicative of incomplete beta oxidation. By 8wks of supplementation, this higher dose exacerbates the skeletal muscle incomplete beta oxidation caused by the HFD, maintains decreased energy expenditure and was unable to ameliorate the HFD-induced IR.

HFD decreases *PGC1α* expression in several tissues, including skeletal muscle [6],[13], which is one of the primary tissues to exhibit IR during the onset of type 2 diabetes [5],[30]. The expression of *PGC1α* is a main determinant of mitochondrial number and function in skeletal muscle in response to environmental stimuli such as dietary inputs [9],[10]. In PGC1α knockout mice, skeletal muscle mitochondrial function is decreased [31]; yet, the mice exhibit increased oxygen consumption, indicative of increased whole body energy expenditure [32]. In the present study, 3wks of a low dose of quercetin increased energy expenditure in the absence of changes in *PGC1α* expression and mitochondrial function. The short term quercetin-induced increases in whole body energy expenditure that precede observed differences in skeletal muscle *PGC1α* expression and mitochondrial function and whole body insulin sensitivity improvements may be due to the pleiotropic effects of quercetin on other tissues, such as liver and adipose [25],[33]. We speculate that, during quercetin supplementation, the lack of coincidence of energy expenditure alterations with insulin sensitivity may also suggest that whole body insulin sensitivity is not likely driven by alterations in whole body energy expenditure alone. Alternatively, changes in energy expenditure may not be sufficient to cause alterations in insulin sensitivity given that alterations in energy expenditure did not result in a change in BW and fat accumulation at the 3wk time point even though food intake was similar between all groups.

Quercetin-induced skeletal muscle *PGC1α* expression increases only after longer supplementation (8wks) of a lower dose (50 ug/day) of supplementation in the present study. This increase parallels changes in acylcarnitine correlation matrices, which show the relationships between acylcarnitine levels and provide us with information on beta oxidation and mitochondrial function. Strong correlations between acylcarnitines within the matrices indicate complete beta oxidation; whereas, reciprocal associations between short and long chain species reflect incomplete beta oxidation that is indicative of reduced mitochondrial function [4],[34]. Thus, the strong positive correlations still present in HF+50Q after 8wks of supplementation but not in HF or HF+600Q, indicate perseverance of mitochondrial function in this group alone. Improved skeletal muscle mitochondrial function coupled with the upregulation of *PGC1α* and increases in whole body insulin sensitivity suggest that quercetin-induced *PGC1α* expression in skeletal muscle may play an important role in determining mitochondrial function and insulin sensitivity. This is supported by the previous report showing that *PGC1α* overexpression in skeletal muscle improves whole body insulin sensitivity by increasing skeletal muscle insulin sensitivity [35], which may be due to changes in skeletal muscle mitochondrial function. Indeed, we show that *PGC1α* expression highly and positively correlates with acylcarnitine levels in skeletal muscle in mice at 3wks, before differences in insulin sensitivity or *PGC1α* expression levels are observed. Our observed correlations may indicate that *PGC1α* expression levels may drive quercetin-induced alterations in mitochondrial function in skeletal muscle [8]–[12],[36], which is recapitulated by quercetin-induced changes in acylcarnitine levels [4],[37],[38]. This finding is reinforced by the lack of positive correlations observed by 8wks in HF and HF+600Q, in which *PGC1α* levels are similar to in HF and decreased in HF+600Q compared to 3wk *PGC1α* values and where incomplete beta oxidation is apparent via pairwise correlation matrices [4],[37],[39],[40]. Under low dose conditions where quercetin increased *PGC1α* expression in the skeletal muscle at 8wks, strong positive correlations were maintained between *PGC1α* expression and acylcarnitine levels despite consumption of the HFD. This upregulation and relationship coincided with maintenance of mitochondrial function, suggesting that 8wks of low dietary quercetin supplementation is sufficient to improve skeletal muscle mitochondrial function through increasing *PGC1α* expression.

In summary, our study demonstrated that the insulin sensitizing effects of dietary quercetin supplementation occur in a time- and dose-dependent manner. The protective benefits of quercetin in preventing HFD-induced IR occurred only with a low dose of quercetin after a longer supplementation period and were associated with increased skeletal muscle *PGC1α* expression and more complete beta oxidation. However, it appeared that quercetin-induced increases in energy expenditure alone were not sufficient to improve insulin sensitivity. We therefore propose that low, chronic dietary quercetin supplementation can attenuate diet-induced IR and improve skeletal muscle mitochondrial function presumably via upregulation of skeletal muscle *PGC1α*.

## Supporting Information

Table S1 Energy Expenditure Means.
(DOCX)
