# Supplementary material for: Dietary Quercetin Supplementation in Mice Increases Skeletal Muscle PGC1α Expression, Improves Mitochondrial Function and Attenuates Insulin Resistance in a Time-Specific Manner
Source: PLoS One. 2014 Feb 21;9(2):e89365. doi: 10.1371/journal.pone.0089365 (PMC3931728; doi:10.1371/journal.pone.0089365)
Supplement: Table S1 — Energy Expenditure Means. (DOCX) [file pone.0089365.s001.docx]

|  |  | **Energy Expenditure (kJ/kg BW/hr)** | | | | | | | | | | |  |
| --- | --- | --- | --- | --- | --- | --- | --- | --- | --- | --- | --- | --- | --- |
|  | **Period** | **HF** | | |  | **HF + 50Q** | | |  | **HF + 600Q** | | |  |
| **3wk** | day | 60.63 | ± | 0.41 | A | 63.38 | ± | 0.43 | B | 55.53 | ± | 0.39 | C |
|  | night | 71.67 | ± | 0.56 | A | 75.48 | ± | 0.55 | B | 66.24 | ± | 0.51 | C |
| **7wk** | day | 55.38 | ± | 0.39 | A | 59.4 | ± | 0.42 | B | 49.01 | ± | 0.48 | C |
|  | night | 64.65 | ± | 0.52 | A | 67.43 | ± | 0.49 | B | 56.61 | ± | 0.53 | C |

Table S1. Energy Expenditure Means

Means ± SEM are shown for energy expenditure values during the day and night periods. Different letters denote significant differences between groups.
